# Identification of mitophagy-related genes in patients with acute myocardial infarction

**DOI:** 10.1186/s41065-025-00424-5

**Published:** 2025-04-26

**Authors:** Ju-Ying Li, Hong-Kui Chen, Yi-Hao Huang, Yu-Peng Zhi, Yue-E Li, Kai-Yang Lin, Chun Chen, Yan-Song Guo

**Affiliations:** 1 Present Address: The First People’s Hospital of Yibin, Yibin, Sichuan Province 644000 China; 2 Shenli Clincal Medical College of Fujian Medical University, Fuzhou, Fujian Province 350000 China; 3 School of Pharmacy, Fujian Medical University, Fuzhou, Fujian Province 350000 China

**Keywords:** Mitophagy, Acute myocardial infarction, Bioinformatic analysis, Machine learning, Gene

## Abstract

Mitophagy is involved in acute myocardial infarction (AMI) process. However, the role of mitophagy-related genes (MRGs) in the AMI process is not well illustrated. We identified MRGs involved in AMI by bioinformatics analysis. The external datasets were employed for the validation of the MRGs, alongside the execution of cellular and animal experiments. Forty-five MRGs were detected, and machine learning identified the top four hub genes, namely *ALDH2*, *ACSL1*, *IL1B*, and *GABARAPL1*. Additionally, an external validation set was used to screen for three diagnostic markers (*ACSL1*, *IL1B*, and *GABARAPL1*) among these hub genes. Immune infiltration analysis revealed changes in the immune microenvironment among patients with AMI. Finally, the significant upregulation of ACSL1, IL1B, and GABARAPL1 in both cellular and animal models was confirmed. The occurrence of mitophagy was observed in the cell model through transmission electron microscopy (TEM). Our study demonstrated that *ACSL1*, *IL1B*, and *GABARAPL1* possess potential biomarkers for AMI.

**Supplementary Information:**

The online version contains supplementary material available at 10.1186/s41065-025-00424-5.

## Clinical trial number

Not applicable. The present study did not encompass a clinical trial.

## Introduction

The prevalence and fatality rates of cardiovascular disorders are increasing, with acute myocardial infarction (AMI) being the most lethal, posing a considerable risk to a patient’s well-being [1]. AMI is a severe continuation of ischemia and hypoxia of myocardial tissue caused by coronary artery occlusion; this can lead to local or extensive cardiomyocyte injury and necrosis, which can cause serious complications such as cardiogenic shock, heart failure and rupture, and sudden cardiac arrest [2]. Therefore, implementation of prompt and an effective reperfusion therapy such as thrombolysis or percutaneous coronary intervention (PCI) plays a crucial role in minimizing the size of AMI and enhancing patient prognosis [3]. However, a considerable number of patients with an unfavorable prognosis attributed to early diagnosis or early PCI treatment still exist; therefore, identifying the early biomarkers for AMI and potential targets for early drug intervention is pivotal in enhancing the prognostic outcomes of AMI.

Recently, mitophagy has been shown to be involved in the regulation of AMI process [4–6]. Mitophagy is an autophagic process that specifically eliminates or degrades impaired mitochondria [7]. Damaged mitochondria fail to efficiently eliminate the reactive oxygen species (ROS) generated by the electron transport chain, resulting in an accumulation of ROS [8]. Excessive ROS levels affect cell proliferation leading to lipid peroxidation, DNA damage, and apoptosis [9]. Numerous cardiovascular diseases, particularly those characterized by elevated energy demands such as heart failure, hypertrophy, AMI, and ischemia-reperfusion (I/R) injury, are strongly correlated with impaired mitophagy [6, 7]. During AMI and I/R, the activation of the mitochondrial permeability transition pore complex (mPTP) compromises mitochondrial function and increases ROS production [5]. This ultimately results in cardiomyocyte death and myocardial injury. The pink2^−/−^mice and Pgam5^−/−^ mice exhibit mitophagy inhibition after AMI, resulting in increased infarct size, aggravated cardiac injury, and decreased cell survival [10]. Thus, targeting mitophagy-related genes could serve as a potential biomarker or therapeutic strategy against cardiovascular disorders.

## Materials and methods

### Data source

We acquired gene expression profile data for GSE66360, GSE61144, and GSE97320 from the gene expression omnibus database (https://www.ncbi.nlm.nih.gov/geo/*).* GSE66360 (AMI = 49, control = 50) and GSE97320 (AMI = 3, control = 3) were obtained using the GPL570 platform ([HG-U133_Plus_2] Affymetrix Human Genome U133 Plus 2.0 array). The GSE61144 dataset (AMI = 14, control = 10) was obtained using the GPL6106 platform (Sentrix Human-6 v2 Expression BeadChip). In this study, 5131 mitophagy-related genes (MRGs) were obtained from the GeneCards database (https://www.genecards.org/*).*

### Screening of differentially expressed genes (DEGs)

We conducted differential analysis using the ‘limma’ R package to identify DEGs between AMI samples (*n* = 49) and control samples (*n* = 50) obtained from the GSE66360 database. DEGs were selected based on a threshold of|log2(fold change, FC)| > 1 and P-value < 0.05. The R packages “ggplot2” (v3.4.4) and “pheatmap” (v1.0.12) were utilized for generating volcano plots and heat maps, respectively, to visualize the expression of DEGs.

### Weighted gene co-expression network analysis (WGCNA)

In this research, we employed the “WGCNA” R package to construct a co-expression network using gene expression levels from 99 samples obtained from GSE66360 as input data, while considering AMI and control as trait data. Initially, outliers were detected by performing sample clustering using the hclust function, where the parameter was set to ‘method = average’ for distance computation. In addition, we determined the optimal soft threshold by approximating a scale-free network. Subsequently, a dynamic shear tree algorithm was utilized to detect the modules, and correlation analysis was applied to identify the modules linked to AMI. AMI-related genes were identified by selecting the modules that showed the highest correlation with the outcome variables based on their Module Membership and Gene Significance within those modules.

### Identification of mitophagy-related genes (MRGs) in AMI

MRGs were derived by intersecting the DEGs, WGCNA, and mitophagy identified from the GSE66360 dataset. The expression of MRGs in both AMI and control groups was assessed using the Wilcoxon test. Subsequently, we employed the R package “clusterprofiler” to conduct Gene Ontology (GO) and Kyoto Encyclopedia of Genes and Genomes (KEGG) analyses of the MRGs, resulting in the identification of the top 10 GO terms and top 15 KEGG signaling pathways.

### Machine learning

RF, SVM-RFE, and LASSO algorithms were applied using the glmnet package to reduce data dimensions. LASSO algorithms were used to identify gene biomarkers for AMI by selecting features from the MRGs of patients with AMI and control groups. The effectiveness of the gene biomarkers was assessed by analyzing the receiver operating characteristic (ROC) curve and calculating the area under the curve (AUC), accuracy, sensitivity, and specificity. In addition, we assessed the diagnostic precision of the logistic regression model using ROC curves from the external GSE61144 and GSE97320 datasets. We employed a well-established risk score algorithm to compute the penalty coefficients for the feature genes and subsequently assigned weights based on individual gene expression values that were normalized.

### Immune infiltration analysis

The immune activity of each sample was accurately determined using 28 immune-related gene sets (http://xteam.xbio.top/CellMarker*).* Initially, the present study employed the “GSVA” R package to compute immune gene set compositions in 49 AMI and 50 control groups. Subsequently, an investigation was conducted to examine disparities in immune gene sets between AMI and control groups. Furthermore, the associations between key genes and differentially expressed immune cells were analyzed, along with correlations between immune cells.

### Animals

C57BL/6J mice used for animal studies were purchased from SLAC (Shanghai, China). All experimental designs and protocols involving animals adhered to the “Guidelines for the Care and Use of Experimental Animals” and received approval from the Animal Ethics Committee of Fujian Medical University. The mice were kept in a controlled environment that was free of pathogens, maintained at a standard temperature range of 20–23 °C, and subjected to a 12-h light-dark cycle. The experimental mice were randomly assigned to groups to ensure that all in vivo experiments and subsequent evaluations were conducted without the knowledge of the groups.

### Animal MI model

Male mice (6–8 weeks) were anesthetized with isoflurane (1–2%), the heart was rapidly extruded through an incision in the left thoracic cavity, and the left anterior descending coronary artery was ligated using a silk suture (5 − 0). White ischemic areas and electrocardiographic changes are important indicators of surgical success. The mice in the control group that underwent sham surgery, underwent the same surgical procedure and did not require coronary artery ligation. The heart tissue samples were collected from both groups after 24 h.

### Cardiomyocyte cell line culture and treatment

The H9C2 cell line of rat cardiomyocytes, obtained from Procell in Wuhan, China, was cultured at 37℃ with 5% CO_2_ in Dulbeccos Modified Eagles Medium (DMEM) supplemented with 10% fetal bovine serum (FBS) and 1% penicillin-streptomycin. To create a hypoxic environment, the incubator gas was comprised of 1% O_2_, 5% CO_2_, and 94% N_2_. H9C2 cardiomyocytes were subjected to these conditions for 12 h to induce hypoxia. In contrast, the control group’s H9C2 cells were continuously cultured under normoxic conditions (21% O_2_, 5% CO_2_, and 74% N_2_).

### Western blot (WB)

The RIPA buffer (Beyotime, China) was utilized to homogenize and lyse the cultured cardiomyocytes as well as the heart tissue located beneath the ligature. The protein (30 µg) underwent separation using an SDS-PAGE gel, followed by transfer onto a polyvinylidene fluoride (PVDF) film. Subsequently, the PVDF film was subjected to blocking with 5% milk and incubated overnight at 4℃ in the presence of the primary antibody. Comprehensive data on the antibodies employed can be found in Additional file 9 (Additional file 1: Table 1). After incubating for 2 h at ambient temperature, the bands were observed using a chemiluminescent system provided by Bio-Rad. The analysis and quantification of western blot band intensity were performed using the Image J software. GAPDH served as internal controls.

### Quantitative real-time PCR (qRT-PCR)

Total RNA was harvested from hypoxic model cells and the control group. (Steady Pure Mag Tissue & Cell RNA Extraction Kit (Accurate Biology, AG21023, China)). Using the Evo M-MLV Mix Kit with gDNA Clean for qPCR (Accurate Biology, AG11728, China), reverse transcription of the extracted total RNA was performed to obtain cDNA. A SYBR Green Premix Pro Taq HS qPCR Kit (Accurate Biology, AG11701, China) was used for amplification. To quantify the mRNA expression levels, β-actin was used as an internal reference for qPCR. Primer sequences used to study these genes are listed in Additional file 10 (Additional file 10: Table 2).

### Transmission electron microscopy (TEM)

The myocardial organelles were observed using TEM. Myocardial cells from both the hypoxic model and control groups were collected and fixed with a pH-neutral electron microscope fixative that consisted of 3% glutaraldehyde, 1.5% paraformaldehyde, and 0.1 M PBS. After dehydration and embedding, samples were sliced into ultrathin sections and stained with uranium acetate and lead citrate. All images were acquired using a transmission electron microscope (Tecnai G2; USA).

### Statistical analysis

All bioinformatics and Pearson’s correlation analyses were performed using R software (version 4.3.1). The data were expressed as mean ± standard error mean (SEM) and subjected to analysis using GraphPad Prism 8.0. The data utilized exhibited a normal distribution. Two experimental groups were compared using an independent sample t-test. Statistical significance was set at *P* < 0.05.

## Results

### Detection of genes with distinct expression patterns in AMI and control groups

**Fig. 1:**
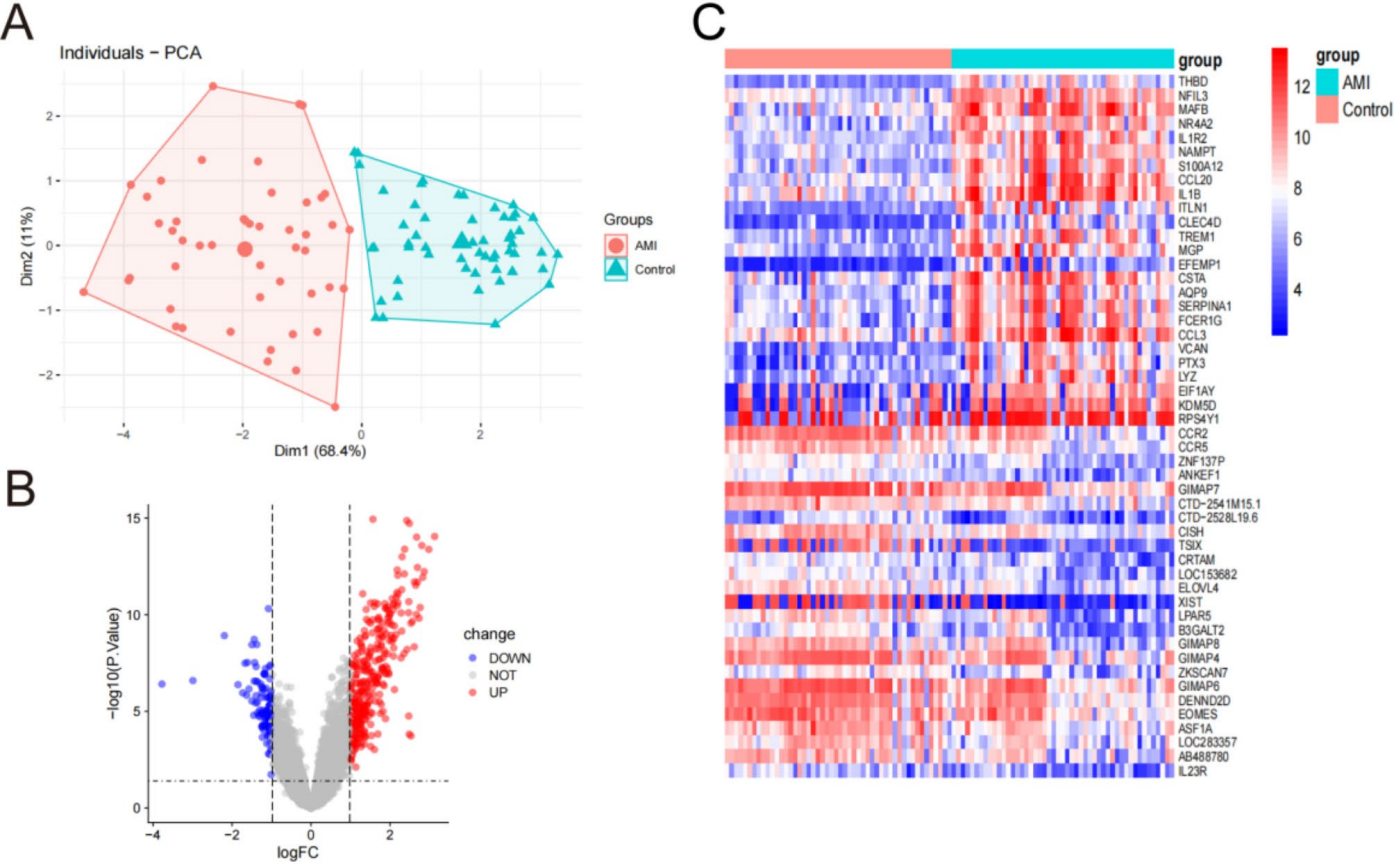
Differentially expressed genes (DEGs) between AMI and control groups. **(A)** Principal Component Analysis (PCA) results. **(B)** Volcano plot of the 441 DEGs. Red dots represent significantly upregulated genes, and blue significantly downregulated genes. (**C**) Heatmap of the 50 DEGs in AMI and control groups

We conducted principal component analysis (PCA) to evaluate the repeatability and discriminative power of MRGs within the group, and the results demonstrated satisfactory performance in both aspects (Fig. 1A). A total of 441 DEGs were identified in the GSE66360 dataset by comparing the AMI and control groups. The DEGs were determined using a cutoff value of *P* < 0.05 and|log2(fold change, FC)| > 1. Among these DEGs, there were 332 upregulated genes and 109 were downregulated (Additional file 3: Table 3). Figure 1B and C display the volcano plots and heat map results for the DEGs.

### Identification of AMI-related genes by weighted gene co-expression network analysis (WGCNA)

**Fig. 2:**
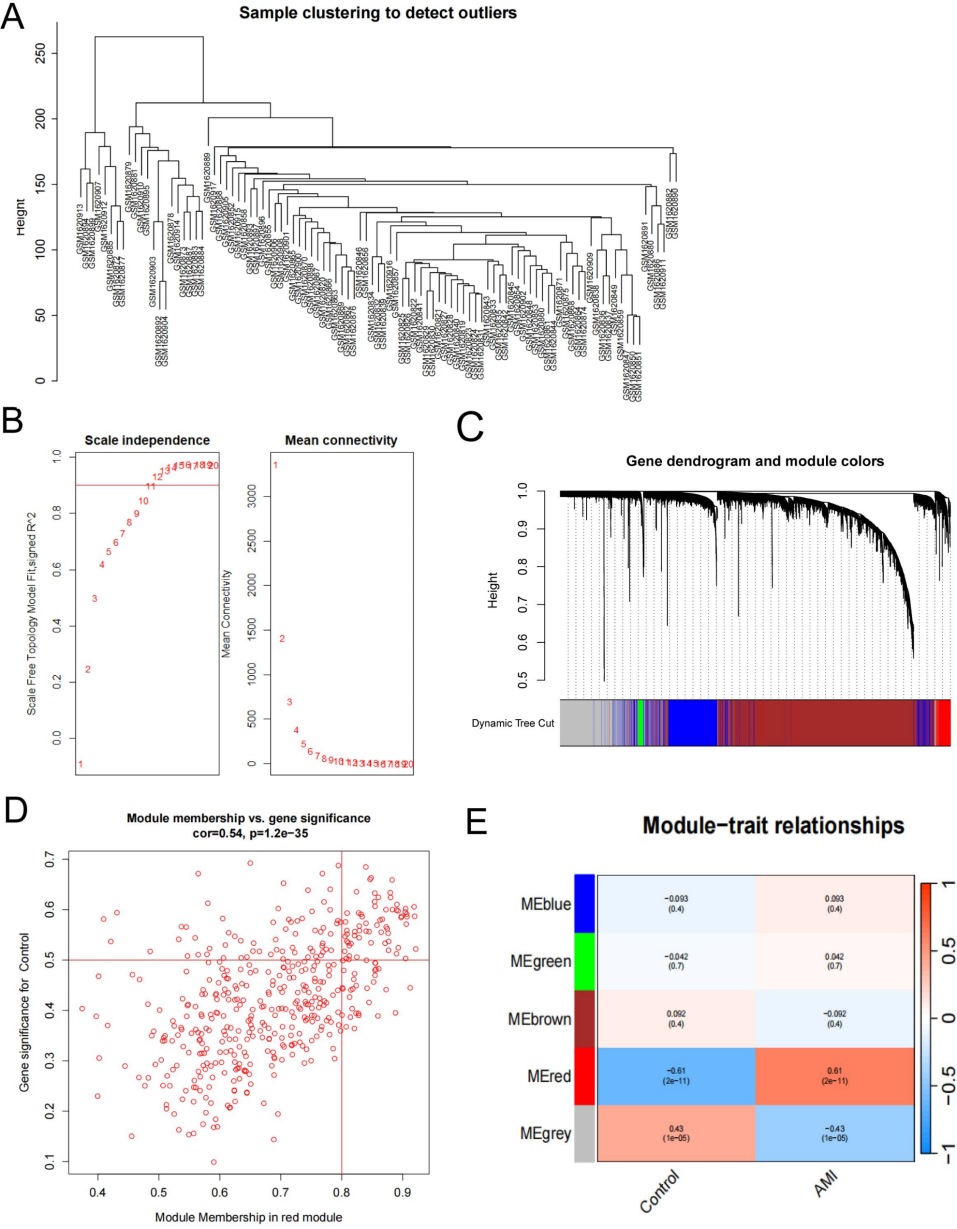
Construction of gene co-expression network in dataset GSE66360. (**A**) Tree diagram of sample clustering. (**B**) The optimal soft threshold value was 11. (**C**) Screening the co-expression module. (**D**) Obtained five modules by dynamic shear tree algorithm. (**E**) Scatter plot of module-trait correlations (red module indicate positive correlations)

First, we set the height cutoff value at 280 and performed clustering analysis based on Euclidean distance to identify promising samples and genes. All samples were included in the subsequent analyses (Fig. 2A). We determined an optimal soft threshold value of 11 from (Fig. 2B) and employed the dynamic shear tree algorithm with a minimum module size of 50 to divide the data into five modules (Fig. 2C and D). Through correlation analysis, we observed a strong association between the red module and AMI, indicating that the genes within this module could be considered AMI-related (Fig. 2E). The specific genes belonging to the red module are provided in Additional file 4: Table 4.

### Identification of MRGs

**Fig. 3:**
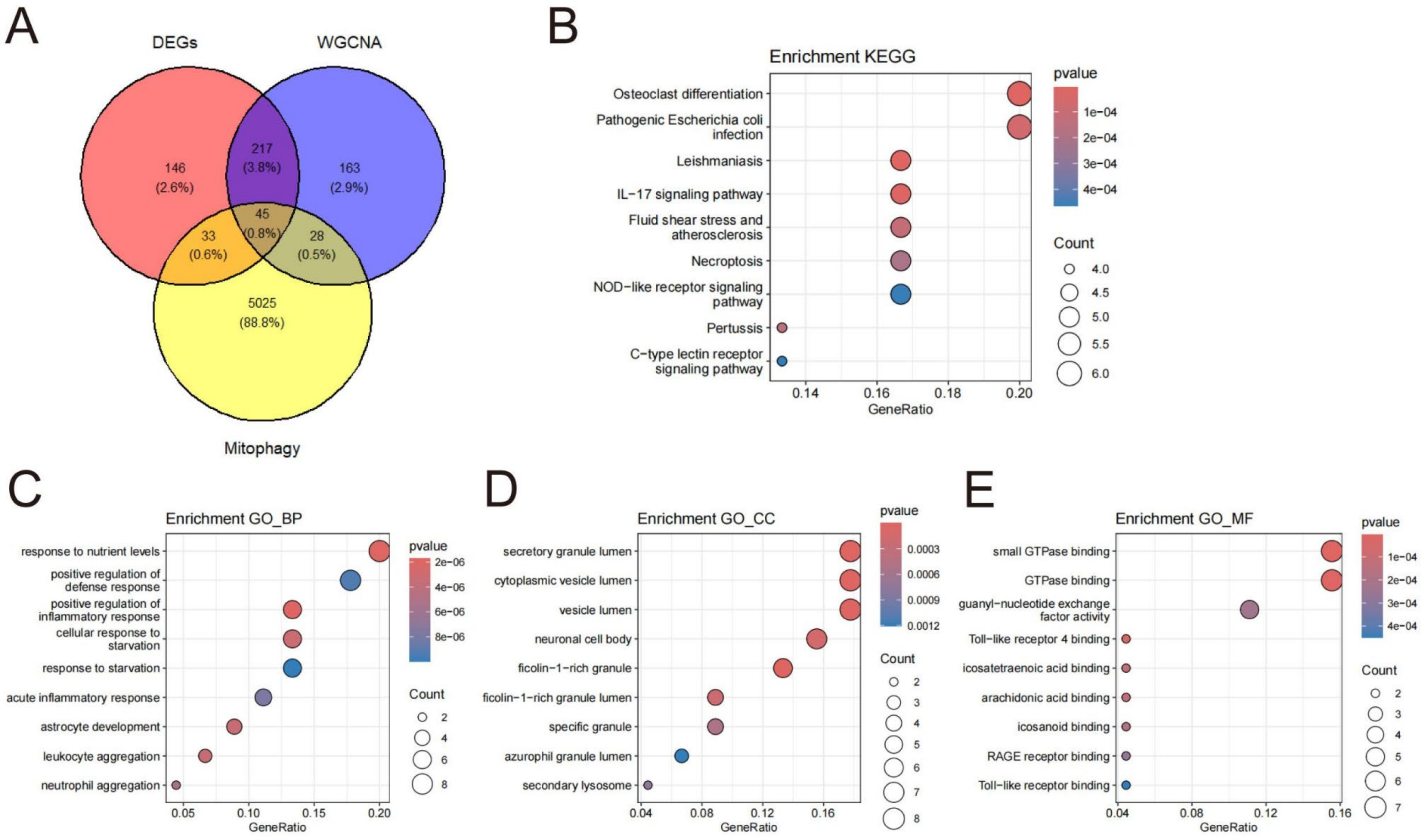
Identification of differentially expressed MRGs in GSE66360 and enrichment analysis of these MRGs. (**A**) Venn diagram of DEGs, WGCNA and Mitophagy. (**B**) Top 15 KEGG pathway. (**B**) Top 10 GO biological processes pathway. (**C**) Top 10 GO cellular component pathways. (**D**) Top 10 GO molecular function pathway

The crossover process resulted in the acquisition of 45 MRGs, as shown in Fig. 3A (Additional file 5: Table 5). We utilized the R package to conduct KEGG pathway and GO enrichment analysis, aiming to demonstrate the biological functionalities of these MRGs. KEGG enrichment analyses revealed that the MRGs primarily focused on pathways associated with infection caused by pathogenic Escherichia coli, the interleukin-17 (IL-17) signaling pathway, atherosclerosis induced by fluid shear stress, necroptosis, NOD-like receptor signaling pathway, C-type lectin receptor signaling pathway, lipid metabolism, atherosclerosis development, and *Salmonella* infection (Fig. 3B). The most enriched GO terms were response to (1) nutrient levels, positive regulation of defense response, regulation of inflammatory response, leukocyte aggregation, response to starvation (biological processes, Fig. 3C); (2) secretory granule lumen, cytoplasmic vesicle lumen, neuronal cell body, mitochondrial outer membrane, ficolin-1-rich granule, specific granule (cellular components, Fig. 3D); and (3) GTPase binding, small GTPase binding, guanyl-nucleotide exchange factor activity, Toll-like receptor binding, icosatetraenoic acid binding, and long-chain fatty acid binding (molecular functions, Fig. 3E). Detailed analyses are available in Additional file 6: Table 6 through Additional file 9: Table S9.

### Identification of key genes

**Fig. 4:**
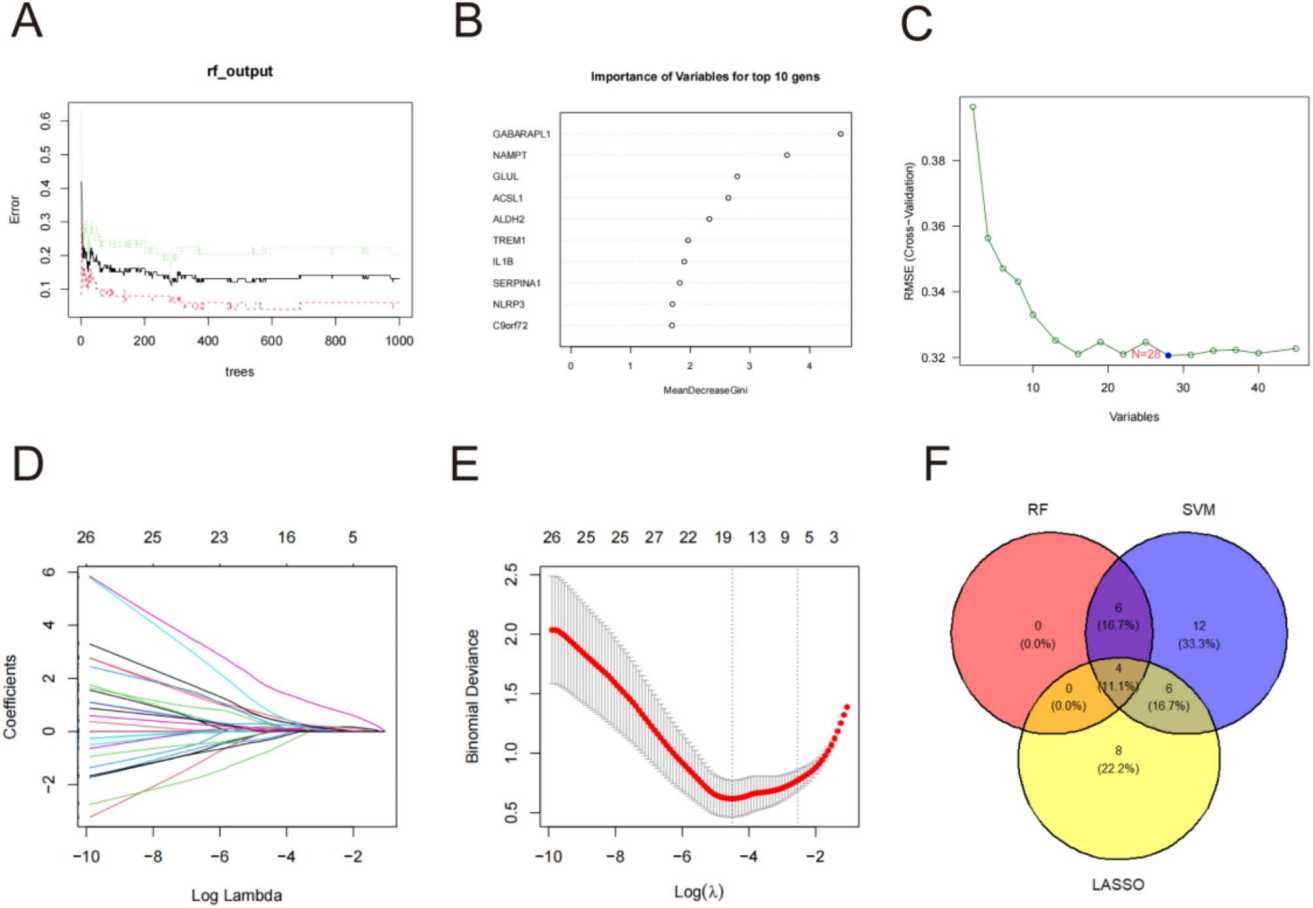
Machine learning models were applied to identify hub MRGs (ALDH2, ACSL1, IL1B and GABARAPL1). (**A**, **B**) The genes were selected based on the random forest (RF) model. (**C**) The support vector machine-recursive feature elimination (SVM-RFE) was used to select the genes. (**D**, **E**) Least absolute shrinkage and selection operator (LASSO) algorithm was used to filtrate the genes. (**F**) Four hub MRGs were identified through the intersection of RF, SVM-RFE, and LASSO models

**Fig. 5:**
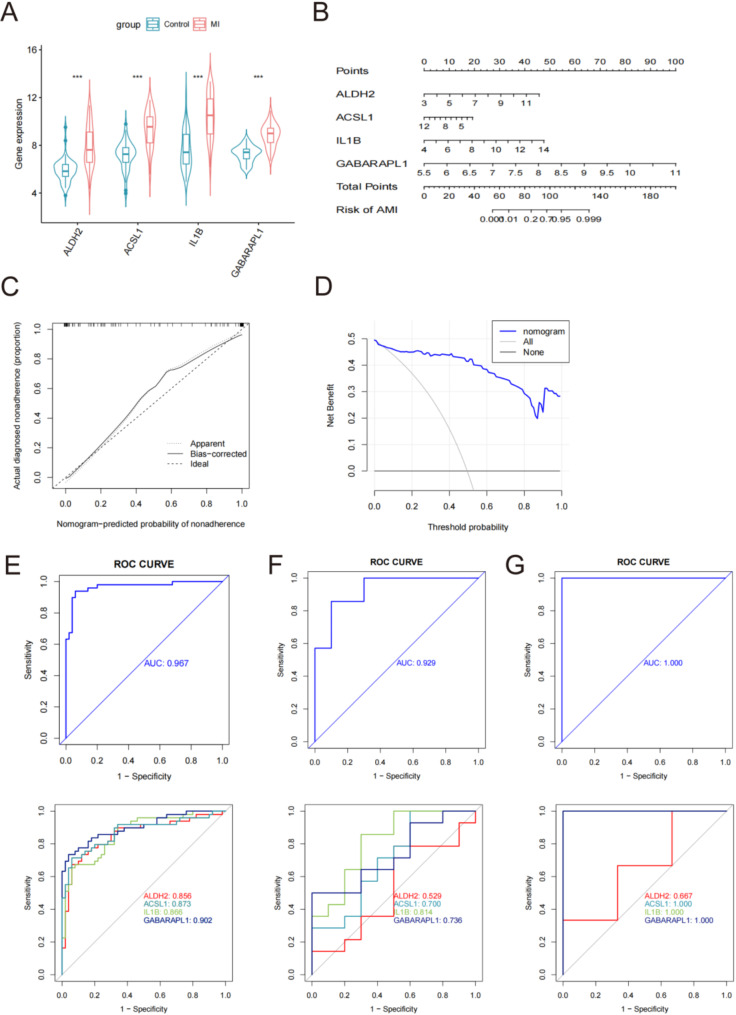
Construction and validation of the AMI diagnostic column line graph. (**A**) The expression levels of these hub genes in the AMI group than control group. (**B**) Diagnostic column line graph was used to predict the occurrence of AMI. (**C**) Calibration curve to examine the predictive power of the diagnostic column line graph. (**D**) Decision curve analysis (DCA) to evaluate the predictive power of the diagnostic column line graph. (**E**, **F** and **G**) Receiver operating characteristic (ROC) curves for evaluating the diagnostic values of the diagnostic column line graph in the test (GSE66360) and validation cohorts (GSE61144 and GSE97320), as well as assessing the diagnostic values of ALDH2, ACSL1, IL1B and GABARAPL1

We employed Random Forest (RF), Support Vector Machine-Recursive Feature Elimination (SVM-RFE), and Least Absolute Shrinkage and Selection Operator (LASSO) regression analyses to screen the 45 MRGs (Fig. 4A-E). The intersection of the RF, SVM-RFE, and LASSO models successfully identified four key genes: ALDH2, ACSL1, IL1B, and GABARAPL1 (Fig. 4F). The MRGs identified by the machine-learning algorithm are listed in Additional file 10 of Table S10. The AMI group exhibited significantly higher expression levels of key genes than the control group (Fig. 5A). A line graph using the RMS package depicted the diagnostic column for EMs (Fig. 5B). The calibration curve showed minimal deviation between predicted and actual risks, underscoring model accuracy (Fig. 5C). Decision curve analysis indicated that the diagnostic column line graph provided a superior net clinical benefit over other strategies (Fig. 5D). These hub genes exhibited excellent diagnostic efficacy for identifying AMI (AUC = 0.967) (Fig. 5E). Among them, *GABARAPL1* exhibited the highest diagnostic value (AUC = 0.902). The remaining genes exhibited the following AUC: *ACSL1* (AUC = 0.873), IL1B (AUC = 0.866), and *ALDH2* (AUC = 0.856) (Fig. 5E). We utilized GSE61144 and GSE97320 as external validation datasets to assess the effectiveness of the hub genes. The outcomes demonstrated remarkable performance, with AUC values of 0.7 and 1 respectively (Fig. 5F and G). All genes, except for *ALDH2*, exhibited AUC areas exceeding 0.7 (Fig. 5F and G). These results suggest that *GABARAPL1*,* ACSL1*, and *IL1B* are potential diagnostic biomarkers for AMI.

### Immune cells score analysis in AMI and control groups

**Fig. 6:**
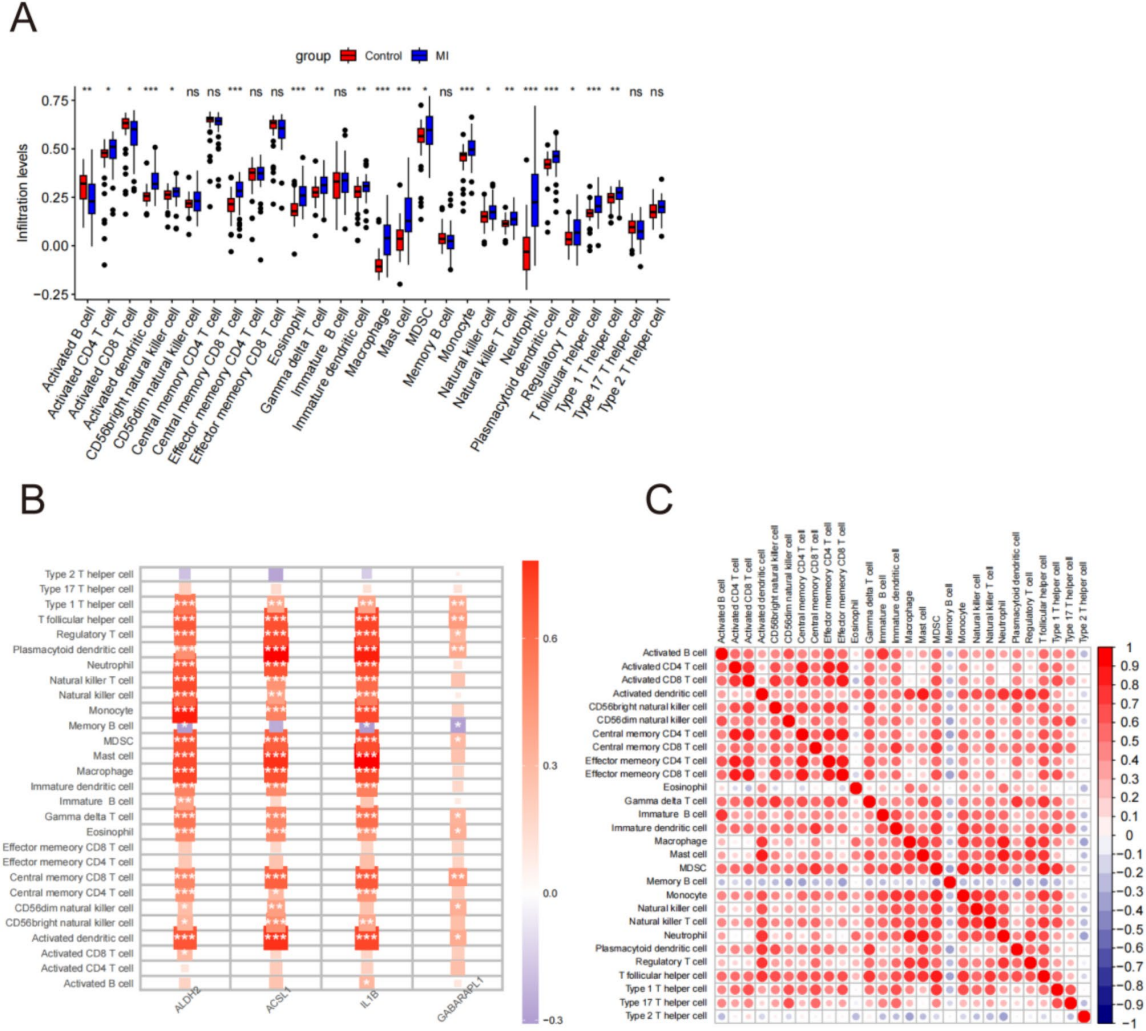
Analysis of immune cells infiltration and correlations between the hub genes and the differential immunity cells, as well as the correlations between immune cells. (**A**) Showing the distribution of 28 immune cells in AMI and control samples of the GSE66360. (**B**) The correlations between the hub genes and the differential immunity cells. (**C**) The correlations between immune cells. *P < 0.05; **P < 0.01; ***P < 0.001, ns (*p* > 0.05)

Figure 6A shows a box-line plot illustrating the expression levels of the 28 immune cell sets across all samples. The AMI group exhibited an elevated expression of various immune cell types, including activated CD4 + T cells, activated dendritic cells, CD56 bright natural killer (NK) cells, central memory CD8 + T cells, eosinophils, gamma delta T cells, immature dendritic cells, macrophages, mast cells, MDSC, monocytes, NK-T cells, neutrophils, plasmacytoid dendritic cells, regulatory T cells, T follicular helper cells, and type 1 T helper cells. Conversely, the AMI group exhibited reduced numbers of activated B-cells and CD8 + T cells. Furthermore, Fig. 6B shows a correlation plot depicting the key genes and differential immune gene sets. The levels of *ALDH2*,* ACSL1*, and *IL1B* were positively correlated with various immune cells, including type 1T helper cells, T follicular helper cells, regulatory T cells, plasmacytoid dendritic cells, neutrophils, NK-T cells, NK cells, monocytes, MDSC, mast cells, macrophages, immature dendritic cells, gamma data T cells, central memory CD8 T cells, eosinophils, and activated dendritic cells. The expression levels of *GABARAPL1* were positively correlated with type 1T helper cell, T follicular helper cells, regulatory T cells, plasmacytoid dendritic cells, gamma data T cells, eosinophils, and central memory CD8 T cells. The levels of *GABARAPL1*,* ALDH2*, and *IL1B* were negatively correlated with memory B cells. Furthermore, the analysis of immune cell correlations revealed that memory B cells were negatively associated with other immune cell types (Fig. 6C).

### Validated the identified hub genes

**Fig. 7:**
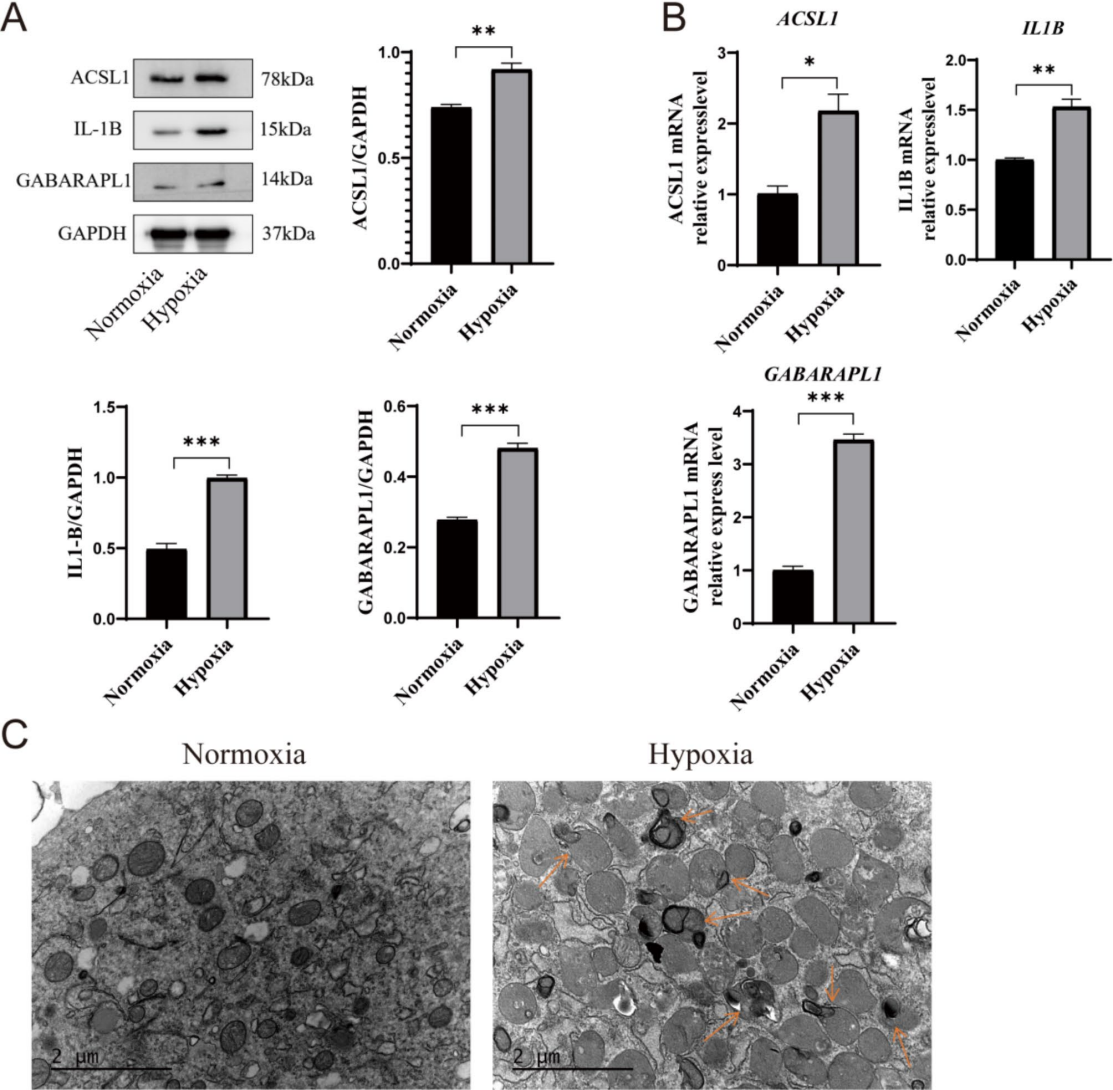
The expression patterns of these central genes and organelles in the cellular experiment. (**A**) In the hypoxic cell model, there is an increase in the protein expression levels of ACSL1, IL1B and GABARAPL1 when compared to the normoxia group (*n* = 3). (**B**) The mRNA expression levels of ACSL1, IL1B and GABARAPL1 exhibit higher magnitudes in hypoxic cell model than normoxia group (*n* = 3). (**C**) The transmission electron microscopy (TEM) analysis revealed an increase in mitophagy in the hypoxic cell model (*n* = 3). Data are presented as mean ± standard error mean (SEM). *P < 0.05, **P < 0.01, ***P < 0.001

**Fig. 8:**
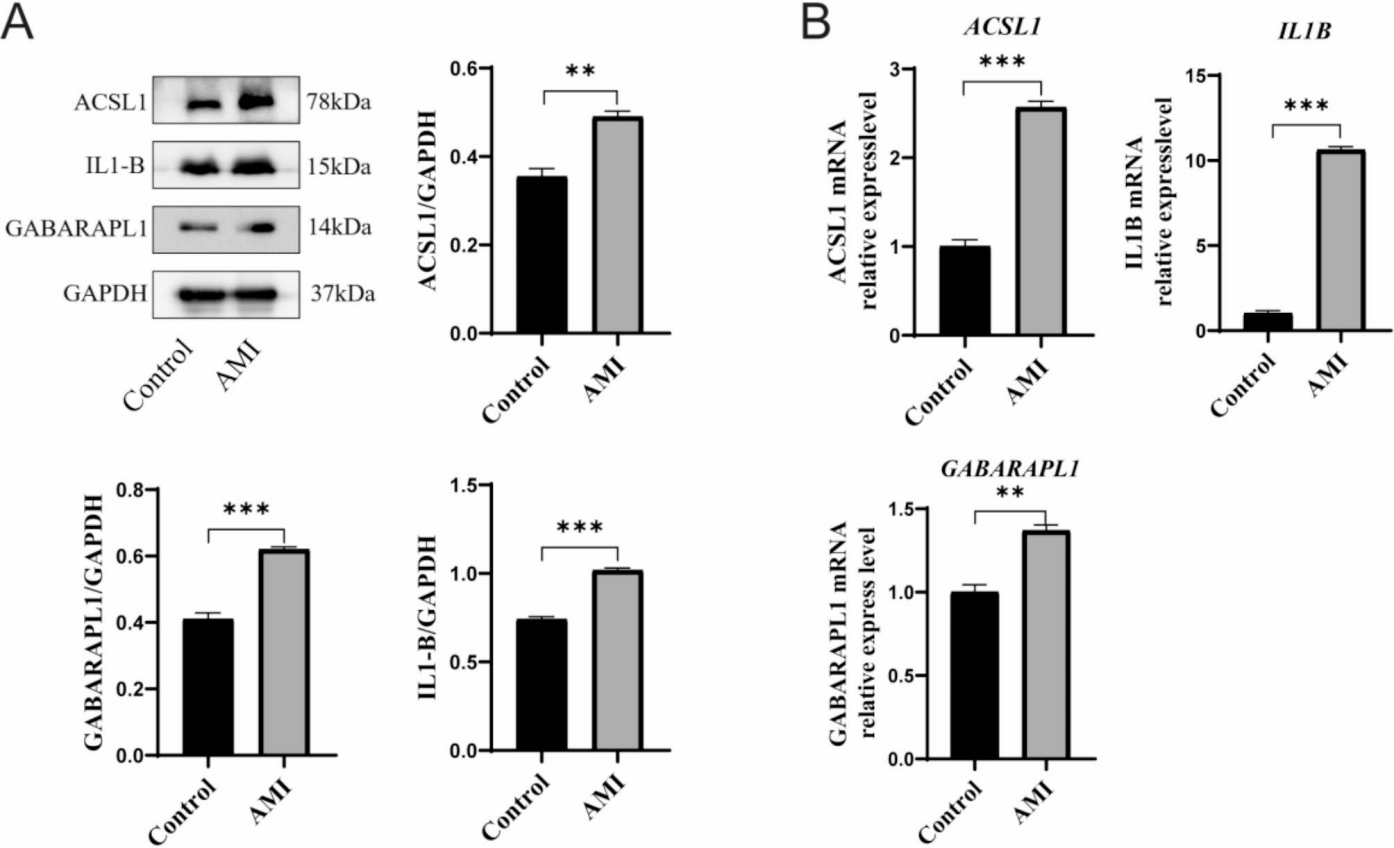
The expression patterns of these hub genes in animal experiments. (**A**)The expression levels of ACSL1, IL1B and GABARAPL1 in AMI animal model were up-regulated in the AMI animal model compared to the control group (*n* = 3). (**B**) The AMI animal model shows an increase in the mRNA expression levels of ACSL1, IL1B, and GABARAPL1 compared to the control group (*n* = 3). All data were represented as mean ± standard error mean (SEM). *P < 0.05, **P < 0.01, ***P < 0.001

Finally, we confirmed the hub genes identified using WB and RT-qPCR. The findings revealed a significant upregulation in the protein and mRAN expression levels of *ACSL1*, *IL1B*, and *GABARAPL1* in both the hypoxic cell model and myocardial infarction animal model compared to the control group (Figs. 7A and B and 8A and B). The hypoxic cell model group demonstrated an increase in lysosomes and autophagosomes, while exhibiting a decrease in mitochondria, as observed by TEM (Fig. 7C).

## Discussion

Acute myocardial infarction (AMI), also known as heart attack, is responsible for the majority of sudden cardiac deaths globally and has the highest fatality rate compared to other cardiovascular conditions. Hence, early detection and intervention are critical for enhancing quality of life and reducing mortality in patients with AMI. Although many mechanistic studies on AMI development have been conducted, our understanding of the mechanism of mitophagy in AMI is limited. However, the complete identification of mitophagy-related genes associated with AMI remains elusive. Therefore, understanding the significance of mitophagy-related genes for the diagnosis and treatment of AMI is imperative.

This study aimed to identify previously unidentified genes associated with mitophagy in patients with AMI. We obtained the GSE66360, GSE61144, and GSE97320 datasets related to AMI and conducted a comprehensive bioinformatics analysis. We analyzed the GSE66360 dataset to detect DEGs among the AMI genes associated with mitophagy. The “limma” R package and enrichment analyses of GO and KEGG pathways were utilized for this purpose. Machine learning techniques have been employed for extensive screening and diagnostic identification of molecular markers linked to AMI. Four MRGs (*ALDH2*,* ACSL1*,* IL1B*, and *GABARAPL1*) were identified as hub genes using various analytical approaches. ROC analysis was conducted on the external datasets GSE61144 and GSE97320 to assess the diagnostic significance of these MRGs in AMI. We performed immune infiltration analysis and analyzed the associations between hub genes and distinct immune genes. These MRGs were also validated in a hypoxic cellular model. Our research aimed to investigate the molecular mechanisms underlying mitophagy in AMI with the ultimate goal of providing guidance for personalized diagnosis and treatment strategies for AMI.

ALDH2 is a type of aldehyde oxidase found in mitochondria and is mainly distributed in organs with high mitochondrial content, including the liver, heart, lung, and kidney [11]. Multiple research studies indicate that the role of ALDH2 could have a beneficial impact on cardiovascular well-being [12]. Enhancing ALDH2 activity through pharmacological means following a myocardial infarction may lead to an improvement in ischemic heart injury [13, 14]. The overexpression of ALDH2 safeguarded the myocardium against I-R injury through the reduction of 4HNE protein adducts, suggesting enhanced 4HNE detoxification mediated by ALDH2 [15]. However, our study revealed that the AUC value of ALDH2 was less than 0.7 during the validation of the external dataset. Consequently, ALDH2 was excluded from the list of core genes associated with mitophagy.

ACSL 1, the initial enzyme in the fatty acid β-oxidation pathway, assumes a crucial function in governing the entry of fatty acids into either synthetic or oxidation pathways [16]. ACSL 1 is positioned within the endoplasmic reticulum, mitochondria-associated membranes and cytoplasmic matrix, directing fatty acids to mitochondria by adding coenzyme A for β -oxidation [17]. Overexpression of ACSL 1 in the mouse heart increases triglyceride accumulation in cardiomyocytes 12-fold [18]. Previous research has indicated a strong correlation between ACSL activity and alterations in hepatic cholesterol and free cholesterol levels in the activity of ACSL [19]. Earlier studies revealed an elevation in the expression of ACSL 1 in the peripheral leukocytes of patients with AMI compared to individuals without any health issues [20]. Li et al. demonstrated that the overexpression of ACSL 1 significantly increased apoptosis in cardiomyocytes [21]. However, knockdown of ACSL 1 reinitiates the cell cycle in cardiomyocytes in vitro and *in vivo.* [22] These results corroborate the conclusions drawn from our bioinformatics analyses and experimental investigations.The expression and activity of ACSL1 are related to myocardial ischemia-reperfusion injury. By regulating fatty acid metabolism in cardiomyocytes, ACSL1 may help reduce myocardial damage and improve outcomes in patients with AMI.

Interleukin-1β (IL-1β) is a highly potent inflammatory cytokine primarily released by monocytes and macrophages, dendritic cells (DC), neutrophils, B lymphocytes and NK cells, and keratinocytes, In response to the Toll-like receptor (TLR), activated complement components, other cytokines and IL-1β itself [23–25]. The occurrence and progression of AMI are significantly influenced by the immune system. Patients with AMI exhibit elevated levels of IL-1β, indicating that activation of the NLRP3 inflammasome pathway and subsequent production of IL-1β play crucial roles in the development of AMI [26]. IL-1β is also responsible for promoting the production of inflammatory substances linked to the development of atherosclerotic plaques such as C-reactive protein and IL-6 [27]. The IL-1β inhibitor canakinumab significantly reduced the first major adverse cardiovascular event and residual inflammatory risk in patients with previous myocardial infarction (MI) [28]. Activation of the NLRP3 inflammasome leads to the production of IL-1β, and notably, the suppression of NLRP3 inflammasome hyperactivation in macrophages is facilitated by promoting mitophagy through the involvement of early endosomal machinery [29]. This observation aligns with our research findings, indicating an elevation of IL-1β in AMI, suggesting its potential involvement in mitophagy. IL1β is a key cytokine in the inflammatory response, and its levels are significantly elevated in AMI patients and associated with myocardial damage and poor prognosis. By blocking IL-1β signaling, IL1β inhibitors reduce inflammatory responses, potentially reducing myocardial damage and improving outcomes in patients with AMI.

GABARAPL1 is a member of the protein family known as γ-aminobutyric acid receptor-associated proteins (GABARAPs), which are associated with lysosomal degradation, autophagy, and the trafficking of intracellular receptors [30]. Under conditions of hypoxia or other stresses, the expression of GABARAPL1 is stimulated in muscle cells or cardiomyocytes, indicating that cells utilize GABRAPL1 as a universal mechanism to respond to stress [31–33]. Recent studies indicated that GABARAPL1 may be involved in selective forms of autophagy [34, 35]. GABARAP1 has been previously linked to selective autophagy in the mitochondria. GABARAP1 interacts with BNIP3L, a mitochondrial protein, and is recruited to mitochondria during reticulocyte maturation to facilitate clearance [36]. The FOXO 3 / GABARAPL1 signaling pathway plays a key role in excessive autophagy/mitophagy, leading to mitochondrial dysfunction in skeletal muscles [37]. Furthermore, our bioinformatics analysis revealed significant upregulation in the expression of GABARAPL1, which was further supported by in vitro and in vivo experimental results.

Research has revealed that tissue damage caused by AMI may manifest within just one hour after the event and activate an inflammatory signaling pathway to trigger an immune response; macrophages are one of the most active cell types in all stages after AMI [38]. After AMI, the damaged heart tissue can trigger a cascade of inflammatory cells and mediators in subsequent waves [39, 40]. The initial phase of inflammation is primarily characterized by the infiltration of polymorphonuclear neutrophils into the myocardium [41]. In the second stage, the main features are macrophage recruitment and initial neutrophil infiltration, and inflammation after AMI exhibits some protective effects [42]. Following AMI, T cells are activated in the mediastinal lymph nodes, which drain from the heart and occur early, along with macrophages. This could be attributed to the presence of autoantigens generated by the release of intracellular proteins during cardiac injury [43]. New findings indicate that natural killer (NK) cells, a diverse collection of innate lymphoid cells, may play a role in the inflammatory response within the heart following AMI [44]. While NK cells may engage with M1 macrophages and promote inflammation, their presence appears to exert a significant safeguarding effect [45]. Mitophagy is increasingly being acknowledged as a key process for regulating the immune system. It accomplishes this by directly controlling mitochondrial antigen presentation and preserving the equilibrium of immune cells [46]. Research has shown that when immune cells experience mitochondrial dysfunction, it can intensify the inflammatory reaction and hinder the repair process after a myocardial infarction [47]. In KEGG and GO enrichment analyses, these MRGs were enriched in the infection and inflammatory response pathways. Immune cell infiltration analysis revealed the presence of macrophages, eosinophils, mast cells, monocytes, NK cells, neutrophils, regulatory T cells, and helper T cells in acute myocardial infarction. Finally, the four hub MRGs were positively correlated with these immune cells. Furthermore, we demonstrated that lysosomes and autophagosomes increased in cardiomyocytes under hypoxia by cell experiments in vitro, with a decrease in mitochondria. Three MRGs diagnostic markers (*ACSL1*,* IL1B*, and *GABARAPL1*) were validated based on their elevated expression levels in the hypoxic group. Therefore, we speculated that these markers in AMI might initiate a series of inflammatory responses during AMI by regulating mitophagy.

However, the current study has certain limitations. First, we conducted bioinformatics analysis using datasets from circulating endothelial cells and validated our findings using animal AMI and hypoxic cardiomyocyte models. Although there may be variations in the samples, our study, similar to other studies utilizing the GSE66360 dataset [48, 49], produced favorable outcomes that strongly support our findings. Furthermore, our study solely encompassed validation in cellular and animal models without the inclusion of clinical samples. We intend to assemble a comprehensive collection of clinical samples for validation and conduct in-depth research. Third, further studies are required to investigate the mechanisms underlying mitophagy in AMI using cellular or animal models.In this study, the key genes involved in mitochondrial autophagy associated with AMI were identified through bioinformatics analysis. These genes, including ALDH2, ACSL1, IL1B and GABARAPL1, have potential applications in the diagnosis and treatment of AMI, providing a strategic approach for personalized treatment of AMI.

## Conclusion

In conclusion, bioinformatic analysis and machine learning techniques validated the involvement of three genes associated with mitophagy in AMI. In machine learning methods, RF can process high-dimensional data and evaluate the importance of variables, LASSO regression selects important features through regularization, and SVM-RFE combines classification performance with feature selection, which can improve the accuracy and robustness of hub gene identification.These genes potentially affect AMI development by regulating autophagy. Our findings contribute to the understanding of the mechanisms linked to mitophagy in AMI and potentially offer novel therapeutic approaches for reducing AMI size and mortality rates.

## Electronic supplementary material

Below is the link to the electronic supplementary material.

Supplementary Material 1

Supplementary Material 2

Supplementary Material 3

Supplementary Material 4

Supplementary Material 5

Supplementary Material 6

Supplementary Material 7

Supplementary Material 8

Supplementary Material 9

Supplementary Material 10

Supplementary Material 11

Supplementary Material 12

Supplementary Material 13

Supplementary Material 14

Supplementary Material 15

Supplementary Material 16

## Data Availability

No datasets were generated or analysed during the current study.

## Abbreviations

AMI: Acute myocardial infarction
MRGs: Mitophagy-related genes
GEO: Gene Expression Omnibus
GO: Gene ontology
KEGG: Kyoto Encyclopedia of Genes and Genomes
RF: Random forest
SVM-RFE: Support vector machine-recursive feature elimination
LASSO: Least Absolute Shrinkage and Selector Operation
ROC: Receiver operating characteristic
qRT-PCR: Quantitative real-time polymerase chain reaction
ALDH2: Aldehyde Dehydrogenase 2
ACSL1: Acyl-CoA Synthetase Long-Chain Family Member 1
IL-1B: Interleukin 1β
GABARAPL1: GABA type A receptor associated protein like1
TEM: Transmission electron microscopy
PCI: Percutaneous coronary intervention
ROS: Reactive oxygen species; I/R: ischemia-reperfusion
I/R: Ischemia-reperfusion
mPTP: Mitochondrial permeability transition pore complex
DEGs: Differentially expressed genes
WGCNA: Weighted gene co-expression network analysis
DCA: Decision curve analysis
AUC: Area under curve
DC: Dendritic cells
NK: Natural killer cells
TLR: Toll-like receptor
NLRP 3: NLR family pyrin domain containing 3
IL-6: Interleukin 6
FOXO 3: Forkhead Box O3
PMNs: Polymorphonuclear neutrophils
