# Supplementary material for: Identification of mitophagy-related genes in patients with acute myocardial infarction
Source: Hereditas. 2025 Apr 26;162:70. doi: 10.1186/s41065-025-00424-5 (PMC12034215; doi:10.1186/s41065-025-00424-5)
Supplement: Supplementary file 16 — Supplementary Material 16 [file 41065_2025_424_MOESM16_ESM.pptx]

## Slide 1
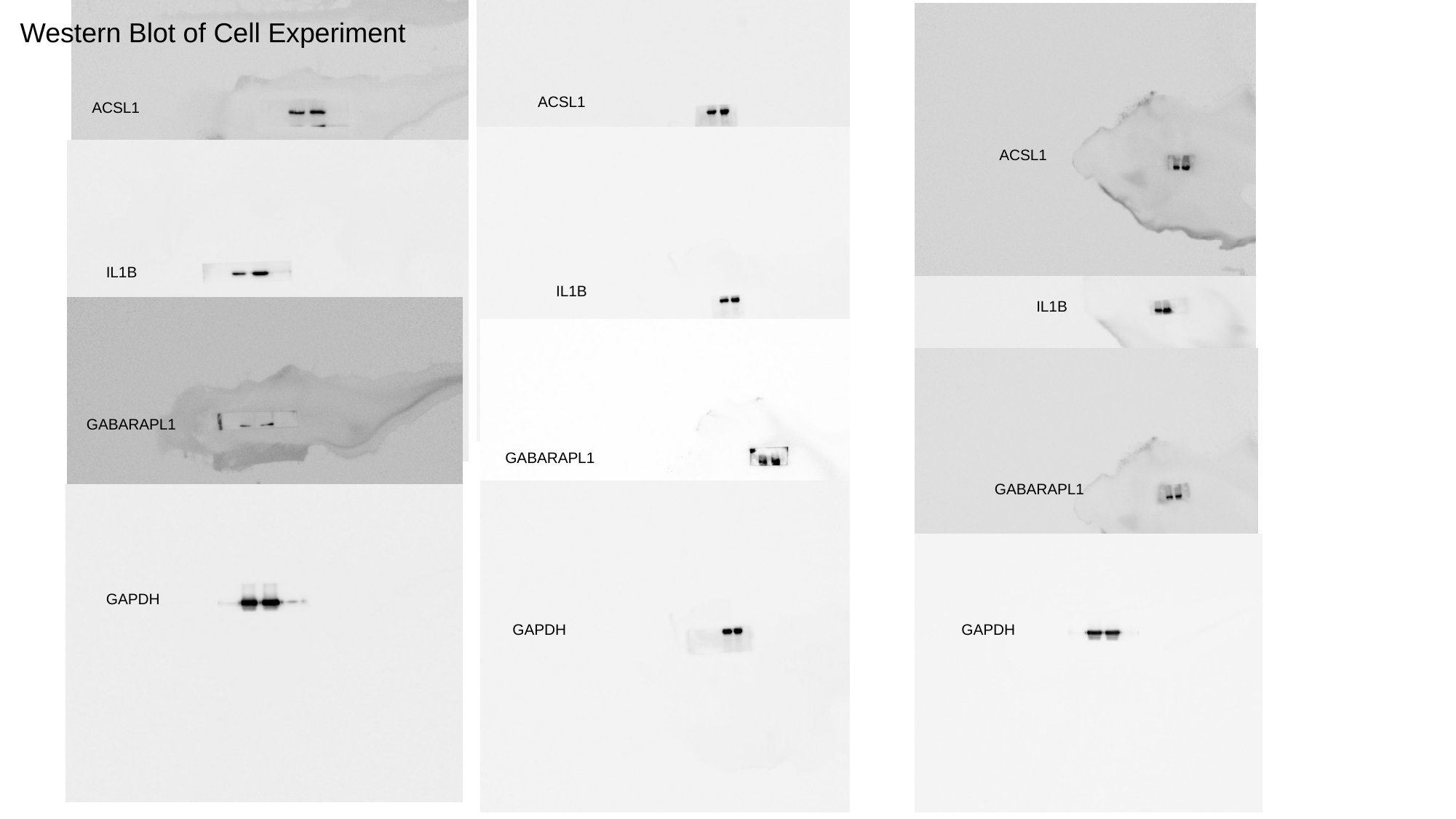

ACSL1
Western Blot of Cell Experiment
ACSL1
IL1B
ACSL1
IL1B
IL1B
GABARAPL1
GABARAPL1
GABARAPL1
GABARAPL1
GAPDH
GAPDH
GAPDH

## Slide 2
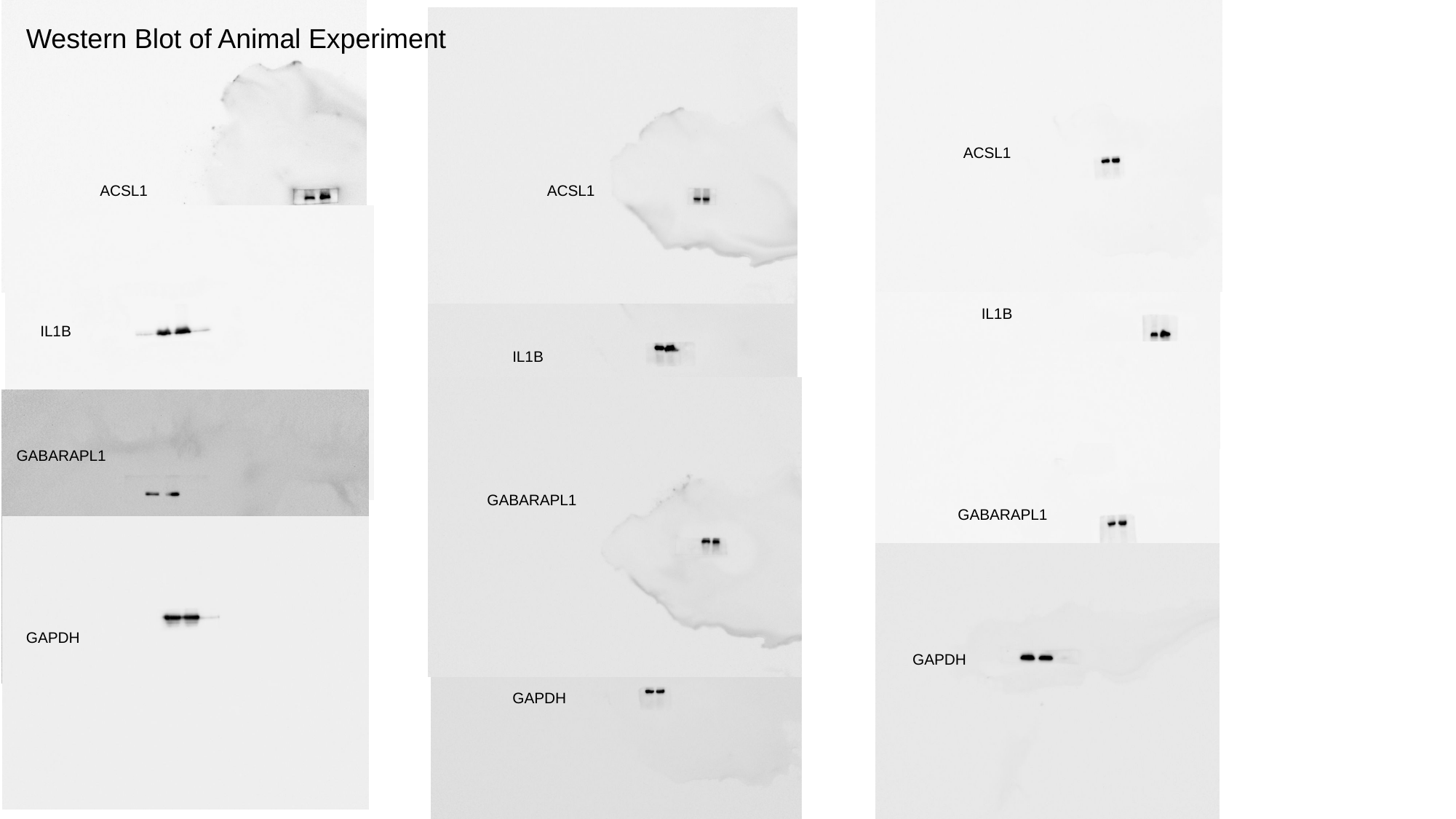

ACSL1
Western Blot of Animal Experiment
ACSL1
ACSL1
IL1B
IL1B
IL1B
GABARAPL1
GABARAPL1
GABARAPL1
GAPDH
GAPDH
GAPDH
